# The Determinants of Traditional Medicine Use in Northern Tanzania: A Mixed-Methods Study

**DOI:** 10.1371/journal.pone.0122638

**Published:** 2015-04-07

**Authors:** John W. Stanifer, Uptal D. Patel, Francis Karia, Nathan Thielman, Venance Maro, Dionis Shimbi, Humphrey Kilaweh, Matayo Lazaro, Oliver Matemu, Justin Omolo, David Boyd, Comprehensive Kidney Disease Assessment for Risk factors, epIdemiology, Knowledge, and Attitudes (CKD AFRIKA) Study

**Affiliations:** 1 Department of Medicine, Duke University, Durham, North Carolina, United States of America; 2 Duke Global Health Institute, Duke University, Durham, North Carolina, United States of America; 3 Duke Clinical Research Institute, Duke University, Durham, North Carolina, United States of America; 4 Kilimanjaro Christian Medical College, Moshi, Tanzania; 5 Tanga AIDS Working Group, Tanga, Tanzania; Centre for Geographic Medicine Research Coast, KENYA

## Abstract

**Introduction:**

Traditional medicines are an important part of healthcare in sub-Saharan Africa, and building successful disease treatment programs that are sensitive to traditional medicine practices will require an understanding of their current use and roles, including from a biomedical perspective. Therefore, we conducted a mixed-method study in Northern Tanzania in order to characterize the extent of and reasons for the use of traditional medicines among the general population so that we can better inform public health efforts in the region.

**Methods:**

Between December 2013 and June 2014 in Kilimanjaro, Tanzania, we conducted 5 focus group discussions and 27 in-depth interviews of key informants. The data from these sessions were analyzed using an inductive framework method with cultural insider-outsider coding. From these results, we developed a structured survey designed to test different aspects of traditional medicine use and administered it to a random sample of 655 adults from the community. The results were triangulated to explore converging and diverging themes.

**Results:**

Most structured survey participants (68%) reported knowing someone who frequently used traditional medicines, and the majority (56%) reported using them themselves in the previous year. The most common uses were for symptomatic ailments (42%), chronic diseases (15%), reproductive problems (11%), and malaria/febrile illnesses (11%). We identified five major determinants for traditional medicine use in Northern Tanzania: biomedical healthcare delivery, credibility of traditional practices, strong cultural identities, individual health status, and disease understanding.

**Conclusions:**

In order to better formulate effective local disease management programs that are sensitive to TM practices, we described the determinants of TM use. Additionally, we found TM use to be high in Northern Tanzania and that its use is not limited to lower-income areas or rural settings. After symptomatic ailments, chronic diseases were reported as the most common reason for TM use which may be particularly important in Northern Tanzania where non-communicable diseases are a rapidly growing burden.

## Introduction

Traditional medicines (TMs) are a critical component of healthcare in sub-Saharan Africa and greater integration between traditional and biomedical health systems may be needed [1–4]. In Tanzania, for instance, alongside increasing access to biomedicine, nearly70% of people still frequently access healthcare through traditional healers or vendors [5, 6]. Because of their importance, understanding how and why people in Tanzania use TMs is necessary.

Others have highlighted the complex dynamics of TM use in sub-Saharan Africa. Cultural belief models, high cost and limited access of biomedicine, disease understanding, safety concerns of biomedicine, and perceptions of TMs as more effective have all been studied as determinants for the use of TM [4, 7–20]. However, TM use among general populations is less well-characterized especially as many of the studies across the region have focused on use in low-income areas, rural settings, healthcare-based samples, or among specific issues such as human immunodeficiency virus (HIV) infection, mental health disorders, bone setting, or midwifery[3, 7, 8,11–13,21–30]. As such, in countries like Tanzania, which has strong historical, cultural, and even political connections to TMs, there remains limited information regarding community-based practices of TMs [31], and studies examining TM users, frequencies of TM use, and determinants for TM use are urgently needed in order to better inform public health efforts [1].

Therefore, we conducted a community-based mixed-method study in Northern Tanzania in order to explore the users of TM, frequency of TM use, and TM practices among the general population. Our overall goal was to describe the determinants of TM use so that we may better formulate local disease prevention and treatment programs that are sensitive to TM practices.

## Methods

### Ethics Statement

The study protocol was approved by Duke University Institutional Review Board (#Pro00040784), the Kilimanjaro Christian Medical College (KCMC) Ethics Committee (EC#502), and the National Institute for Medical Research in Tanzania. The consent forms were administered verbally to all participants, and written informed consent (by signature or thumbprint) was obtained from all participants.

### Study Setting

We conducted a mixed-method study between December 2013 and June 2014 in the Kilimanjaro Region of Tanzania. Our study was conducted in the two districts of Moshi Urban and Moshi Rural. Almost 35%of the regional population lives in urban areas, and it is almost evenly divided between men and women [32]. The adult population includes more than 900,000 people, all of whom access KCMC hospital as their tertiary referral hospital which also serves the neighboring regions. The largest ethnic group is the Chagga tribe followed by the Pare, Sambaa, and Maasai tribes, and Swahili is the major language.

### Qualitative Data Collection

We conducted five focus group discussions (FGDs) composed of 59 participants, and we conducted 27 in-depth interviews, 16 of which were with self-described traditional healers or herbal vendors. We considered the users and providers of both biomedical and traditional healthcare to be key informants. As such, we used purposive sampling to recruit the key informants which included well-adults from the general population, chronically-ill adults receiving care at the hospital medicine clinics, adults receiving care from traditional healers, adults purchasing TMs from herbal vendors, traditional healers, herbal vendors, and medical doctors (MDs) from KCMC hospital. We targeted men and women of all ages from urban and rural settings with different education levels and ethnicities. FGDs were held in a rented office space in Moshi Urban that was well-known and easily accessible to local residents and ensured privacy. Each FGD lasted between four and six hours including breaks. In-depth interviews were conducted at the same office space with the exception of the traditional healers and herbal vendors who were interviewed at their places of work; these sessions lasted one to two hours.

All sessions were semi-structured, open-ended, and probing (S1 Appendix). The discussion guide was initially written in English and then translated to Swahili by an independent team. All sessions were moderated by a native, local member of our team (FK) with the exception of the Maasai healers who were interviewed by a native moderator (ML) in Maa (Maasai language). All sessions were audio-recorded and included two note-takers. Each session was transcribed and independently translated by the note takers (DK and HK) and then reviewed by the moderator to ensure accuracy. Debriefings were held after each session, and team meetings were again held following translation. As important themes emerged, the subsequent sessions were adjusted in an iterative process in order to explore new aspects. We continued this process until we reached data saturation [33, 34].

### Quantitative Data Collection

We developed a structured survey instrument designed to test different characteristics related to TM use and practices in the general population of Kilimanjaro (S2 Appendix). The instrument included open-ended questions related to types of TMs and close-ended questions related to frequency of use, reasons for use, modes of use, modes of access, and conditions treated by TMs.

Local and non-local experts from multiple disciplines were involved in the survey development. After it was constructed in English and was reviewed by the lead investigators (JWS and FK), it was independently translated into Swahili by two native speakers. We then conducted a joint review of each version which included the investigators and translators. Together we explored issues in codability by identifying words and concepts with difficult translations.

To ensure that the survey instrument was of local significance (content validity), we conducted piloting sessions during the FGDs and in-depth interviews. This was an iterative process that involved numerous adjustments to the instrument as new themes and ideas emerged throughout the sessions, and many of the survey items and responses were directly added based on the results of these qualitative sessions.

We then verbally administered the final version of the survey to a random sample of adults from the Moshi Urban and Moshi Rural districts of Kilimanjaro using two trained, local surveyors. For items with multiple response categories, the surveyors were instructed to use direct probing for all the available options.

The sample size was designed to meet the requirements of the CKD AFRIKA project as well as ensure a survey item-to-response ratio of ≥10; therefore, the minimum targeted sample size was 350 individuals. The sampling method was based on cluster probability sampling. Using a random-number generator, thirty seven wards were chosen from the Moshi Urban and Moshi Rural districts based on probability proportional to size. The wards are the most basic government administrative unit in Tanzania, and within each ward a cluster site was determined using geographic points randomly generated using Arc Global Information Systems (ArcGIS), v10.2.2 (Environmental Systems Research Institute, Redlands, CA). Households were then randomly chosen based on coin-flip and die-rolling techniques according to a pre-established protocol (S3 Appendix). To ensure that the protocol was strictly adhered to, the principal investigator (JWS) regularly accompanied the surveyors and conducted anonymous audits.

All adults living in the selected households were recruited, and all Tanzanian citizens over the age of 18 were eligible for inclusion. To reduce non-response rates, we attempted a minimum of two additional visits on subsequent days and weekends, and using mobile phones numbers, we located eligible participants through multiple phone calls. Additionally, when available, we collected basic demographic data for the non-responders.

### Data Analysis

We conducted a thematic analysis of the qualitative data by applying an inductive approach to the framework method [35]. After data reduction, we performed open-coding of all transcripts. We used a ‘cultural insider’ (emic) and a ‘cultural outsider’ (etic) to independently code the data. The cultural insider was a native researcher living in the region (FK) and the cultural outsider was a researcher foreign to the region (JWS). Comparisons were made between each code set and areas of disagreement were discussed and resolved by revisiting the data. This approach allowed us to explore concepts that otherwise may have been overlooked or misinterpreted by either researcher individually.

The qualitative coding, analytic memos, and corresponding matrices were stored and analyzed using NViVOv.10.0 (QRS International Pty Ltd, Melbourne, Australia). The codes were grouped together into categories, and we used a coding index to formulate connections and explore relationships. To ensure that the emerging concepts were cross-checked with other data from all transcripts, we continually refined our analyses in an iterative insider-outsider process. On the basis of the framework method, the major themes were directly derived from this process whereby we used tree maps to visualize relationships among the emerging conceptual categories in order to connect and merge them together to form the larger thematic constructs of our descriptive model [35]. This inductive process helped ensure that the major themes were grounded in the data and the participants’ responses rather than *a priori* assumptions.

Quantitative data were analyzed using STATAv.13 (STATA Corp., College Station, TX). Continuous variables are reported as median (inter-quartile range). All p values are two-sided at a 0.05 significance level. Risk ratios (RR) were calculated by cross-tabulation or by multi-nomial logistic regression in cases of polymatous categorical outcomes. We used Taylor Series linearization to account for the design effect due to cluster sampling. In the final step, the results of the quantitative analyses were triangulated with the qualitative results in order to explore converging and diverging results.

### Data Management

All data were collected on paper and then electronically entered into a purpose-built Research Electronic Data Capture (REDCap) database. All data were verified after electronic data entry by an independent reviewer to ensure accuracy.

## Results

FGDs and in-depth interviews were evenly split among gender (51% male) and had an age range of 18 to 74 years. Most qualitative participants were of the Chagga ethnic group (n = 39; 45%) and were Roman Catholic (n = 31; 36%), but Islamic (n = 20; 23%), Lutheran (n = 19; 22%), Christian evangelical (n = 11; 13%) and Maasai traditional practices (n = 3; 3%) were also represented across thirteen different tribal ethnicities. Education levels varied from none (n = 9; 10%) to university level (n = 13; 15%), but the majority had only completed a primary education (n = 51; 59%)(Table 1). Among the traditional healers and herbal vendors only nine (56%) had received any formal education and none were educated beyond the primary level (Table 2).

**Table 1:** Baseline characteristics of the focus group discussions (FGDs) and in-depth interviews.

	FGD1	FGD2	FGD3	FGD4	FGD5	In-Depth Interviews
Study Population	Clinic Patients	General Population	Clinic Patients	General Population	Medical Doctors	Patients from Healers and Vendors
Participants (N)	15	12	16	12	4	11
Gender						
Male	0%	0%	100%	100%	50%	45%
Female	100%	100%	0%	0%	50%	55%
Age range (years)	25–61	26–65	18–70	18–74	30–36	19–60
Ethnicity						
Chagga	11 (73%)	9 (75%)	11 (69%)	4 (33%)	2 (50%)	2 (18%)
Pare	2 (13%)	2 (17%)	2 (13%)	5 (42%)	0	0
Maasai	0	0	0	0	0	4 (36%)
Sambaa	1 (7%)	1 (8%)	1 (6%)	0	0	3 (27%)
Other*	1 (7%)	0	2 (13%)	3 (25%)	2 (50%)	2 (18%)
Education						
None	0	0	0	0	0	2 (18%)
Primary	11 (73%)	10 (83%)	10 (63%)	3 (25%)	0	4 (36%)
Secondary	3 (20%)	2 (17%)	5 (31%)	6 (50%)	0	1 (9%)
University	1 (7%)	0	1 (6%)	3 (25%)	4 (100%)	4 (36%)
Occupation						
Unemployed^#^	2 (13%)	4 (33%)	0	1 (8%)	0	3 (27%)
Student	0	0	4 (25%)	5 (42%)	0	0
Farmer/Wage Earner	4 (27%)	3 (25%)	8 (50%)	3(25%)	0	5 (45%)
Small Business	3 (20%)	2 (17%)	3 (19%)	2 (17%)	0	1 (9%)
Professional^†^	4 (27%)	3 (25%)	1 (6%)	1 (8%)	4(100%)	2 (18%)
Religion						
Roman Catholic	5 (33%)	5 (42%)	8 (50%)	1 (8%)	3 (75%)	7 (64%)
Lutheran	6 (40%)	4 (33%)	4 (25%)	2 (17%)	0	1 (9%)
Christian Evangelical	1 (7%)	1 (8%)	2 (13%)	5 (42%)	1 (25%)	1 (9%)
Christian (Other)	2 (13%)	0	0	0	0	0
Islam	1 (7%)	2 (17%)	2 (13%)	4 (33%)	0	2 (18%)

*Other Tribal Ethnicities represented in our groups include Luguru, Kilindi, Kurya, Mziguwa, Mnyisanzu, Rangi, Jita, Nyambo, and Kaguru.

^#^ Includes housewives.

^†^ Professional includes any salaried position (e.g. nurse, teacher, government employee, etc.) and retired persons.

**Table 2:** Baseline characteristics of the traditional healers and herbal vendors.

In-Depth Interview (#)	Gender	Age	Ethnicity	Religion	Occupation	Education
1	Male	48	Sambaa	Islam	Traditional Healer	Primary
2	Male	50	Sambaa	Islam	Traditional Healer	None
3	Male	57	Sambaa	Islam	Traditional Healer	Primary
4	Male	65	Sambaa	Islam	Traditional Healer	Primary
5	Male	45	Sambaa	Islam	Traditional Healer	Primary
6	Male	50	Maasai	Lutheran	Herbal Vendor	Primary
7	Male	48	Sambaa	Islam	Herbal Vendor	Primary
8	Male	40	Sambaa	Islam	Herbal Vendor	Primary
9	Male	21	Sambaa	Islam	Traditional Healer	Primary
10	Male	63	Sambaa	Islam	Herbal Vendor	Primary
11	Female	56	Meru	Maasai Traditional	Traditional Healer	None
12	Female	60	Maasai	Roman Catholic	Traditional Healer	None
13	Male	48	Maasai	Maasai Traditional	Traditional Healer	None
14	Male	60	Maasai	Maasai Traditional	Traditional Healer	None
15	Male	56	Maasai	Roman Catholic	Traditional Healer	None
16	Male	50	Maasai	Lutheran	Herbal Vendor	None

Between January and May of 2014, for the quantitative sampling of the structured survey, we enrolled 655 participants from 477 households. The household non-response rate was 15%, and the total sample consisted of 160 men (24.4%) and 495 women (75.6%) with a median age of 43.0 years (33–57). The majority of participants lived in an urban setting (n = 512; 78.2%). The demographics were similar to our qualitative sample with most being Chagga (n = 380; 58.1%), Roman Catholic (n = 258; 39.6%), farmers or daily wage-earners (n = 268; 40.9%), and educated to the level of primary school (n = 481; 73.7%)(Table 3).

**Table 3:** Baseline characteristics of the structured survey respondents.

Variable	Urban (%)(n = 512)	Rural (%)(n = 143)	Total Number (%)(n = 655)
Gender			
Male	123 (24.0%)	37 (25.9)	160 (24.4)
Female	389 (76.0%)	106 (74.1)	495 (75.6)
Age (years)			
18–39	225 (44.0%)	45 (31.5)	270 (41.2%)
40–59	183 (35.7%)	56 (39.1)	239 (36.5%)
60+	104 (20.3%)	42 (29.4)	146 (22.3%)
Ethnicity			
Chagga	302 (59.1%)	78 (54.6)	380 (58.1%)
Pare	55 (10.8%)	35 (24.5)	90 (13.8%)
Sambaa	29 (5.7%)	11 (7.7)	40 (6.1%)
Other	125 (24.4%)	19 (13.3)	144 (22.0%)
Education			
None	31 (6.1%)	4 (2.8)	35 (5.4%)
Primary	356 (69.7%)	126 (88.1)	481 (73.7%)
Secondary	90 (17.6%)	12 (8.4)	102 (15.6%)
Post-Secondary	34 (6.6%)	1 (0.7)	35 (5.4%)
Occupation			
Unemployed	94 (18.4%)	6 (4.2)	100 (15.3%)
Farmer/Wage Earner	159 (31.1%)	109 (76.2)	268 (40.9%)
Small Business	201 (39.3%)	19 (13.3)	220 (33.6%)
Professional	58 (11.3%)	9 (6.3)	67 (10.2%)
Religion			
Roman Catholic	208 (40.9%)	50 (35.2%)	258 (39.6%)
Lutheran	118 (23.2%)	32 (22.5%)	150 (23.0%)
Islam	122 (24.0%)	51 (35.9%)	173 (26.6%)
Other	61 (11.9%)	9 (6.4%)	70 (10.8%)

Reported use of TM is high in Kilimanjaro. Most structured survey participants (67.8%) reported knowing someone who frequently uses traditional medicines, and the majority of participants (55.7%) reported using TMs themselves in the previous year. There was no significant difference in self-reported frequency of use between rural and urban participants (p = 0.75).

**Fig 1:**
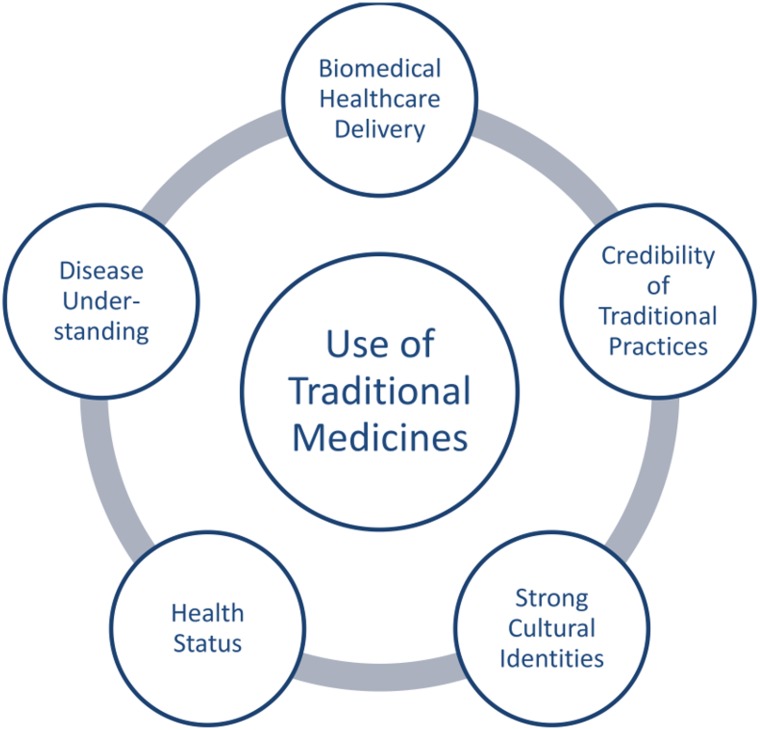
Determinants for the use of Traditional Medicines. The diagram illustrates the five major determinantsand their relationship to each other.

We identified five major determinants for the use of TMs in Kilimanjaro: biomedical healthcare delivery, credibility of traditional practices, strong cultural identities, individual health status, and disease understanding (Fig 1). Together, these determinants describe the factors that influence the uptake of TMs among individuals in Kilimanjaro.

### Biomedical Healthcare Delivery

Structural issues and point-of-care communication issues played major roles in the perceived quality of healthcare delivery. When participants reported receiving low-quality healthcare, there was increased reporting of medical non-compliance, worse medical outcomes, and poorer individual disease understanding which all contributed to increased TM use.

Key structural issues included medication cost, long wait-times, lack of experience and training by some providers, and financial incentives for providers to focus on their private clinics. From the quantitative structured survey most participants (67.1%) agreed that cost is a major factor in the use of traditional medicines, and during the FGDs, a 21 year old male expressed, “*Some doctors are money-oriented*. *If you do not have money then you will never get any service*, *or the service you do get will be very poor*…*”*

All point-of-care issues leading to participants’ reporting of low-quality healthcare delivery were due to ineffective communication between providers and patients. This led to different expectations on behalf of the patient and provider. The qualitative sessions highlighted the role that poor point-of-care communication and healthcare delivery played in these responses. Participants stated that they were concerned about inappropriate or ineffective therapies, and the MDs expressed frustration that their patients had unreasonable expectations of biomedical treatments which they attributed to poor disease understanding:

53yo M—*People complain that if you go to the hospital*, *the first thing they will do is to admit you and start injecting you with a water drip [IV saline] without even testing or explain what the problem is…plus some of the Doctors respond harshly to the patients which makes it hard to believe in them*.

22yo M—*I was admitted to the hospital for three months*, *but I didn’t get any relief*. *The doctors told me that the problem is so serious that is cannot be cured*. *Afterwards*, *my grandmother sent me to the traditional healer*, *and my condition improved in a very short period of time*.

Medical Doctor—*People keep on seeking a cure for something that is a chronic disease*. *For example*, *there was a man who had residual paralysis from a stroke*. *He kept searching for a medicine to cure the paralysis*. *When everything failed*, *he blamed us for not doing enough to cure his paralysis*.

### Disease Understanding

The biologic understanding of disease, including its causes, symptoms, consequences, and treatment, played a fundamental role in the decision to use TMs. Different local disease understandings led to unrealistic expectations of cure, perceived treatment failure, and/or medical non-compliance, which all directly contributed to the use of TMs. Participants’ understandings were a composite interaction among the quality of biomedical healthcare delivery, traditional health belief models, chronicity of the disease, and disease expression through specific symptom complexes.

Structural barriers such as long wait-time and lack of experience and training by some providers prevented participants from receiving education regarding their conditions, and the MDs stressed the difficult role of communicating with patients at the point-of care. One MD stated,

“*Sometimes patients need long-term counseling on the fact that they need medications for life*. *Some react immediately to this by trying alternatives or losing hope*. *It can take much time to get them to understand their diseases and the need for medication*.”

The role of health belief models in disease understanding was most conspicuous for epilepsy and mental health. The pervasive association between these conditions and “evil spirits” led to a nearly ubiquitous use of TMs in these circumstances. However, this was also the case for other conditions such as kidney disease, cancers, diabetes, and malaria irrespective of prior successes or failures with biomedicines. A 30 year old female said, “*I do not take hospital medicines for any disease until I prove that the traditional medicines have failed completely*.*”*

**Fig 2:**
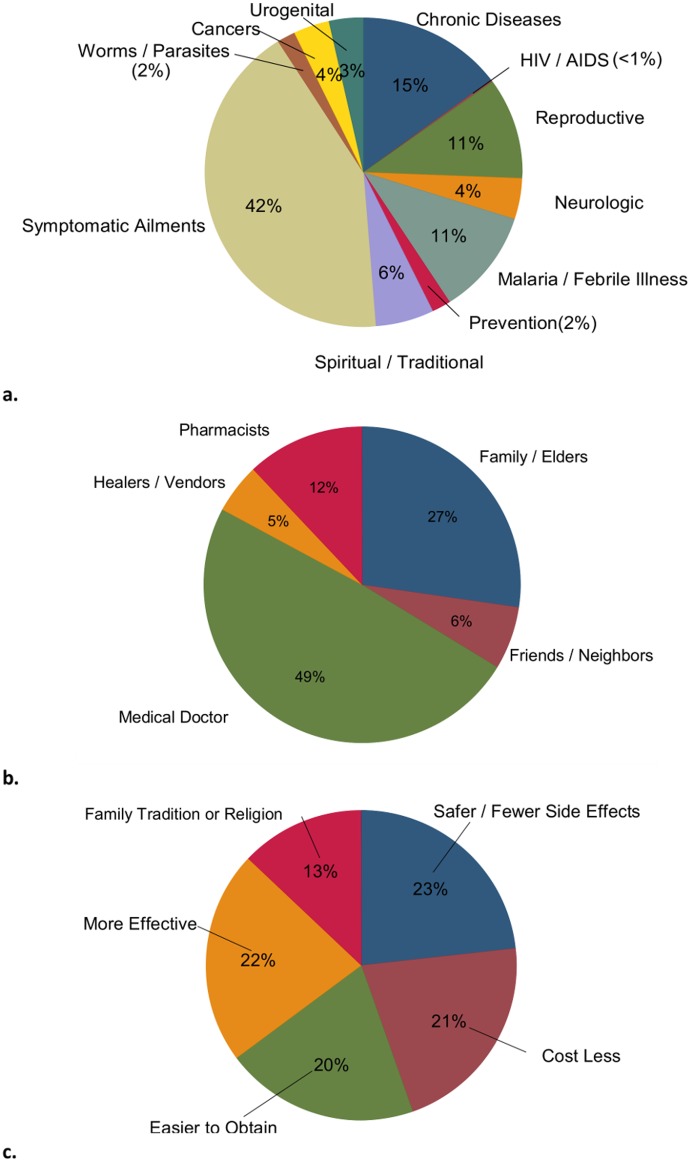
Most common conditions treated by traditional medicines (a)*, means of accessing healthcare (b), and reasons for using traditional medicines (c) among the adult population of Kilimanjaro, Tanzania. * Chronic Diseases: Hypertension, Heart problems, Diabetes, and Body Swelling Reproductive: Sexual Arousal/Virility, Menstrual Problems, Pregnancy Termination, and Fertility/Impotence Neurologic: Epilepsy, Mental Confusion, and Depression Spiritual/Traditional: Peace of mind/Ward off curses, Protection from ‘evil eyes’, Unexplained Illnesses, and To Improve Luck Symptomatic Ailments: Increase Strength, Constipation, Increase energy, Digestion/Stomach problems, Fatigue, Arthritis/joint pains, Flu/Cold symptoms, Headaches, and Skin problems Urogenital: Kidney problems and Urinary problems

Many participants had a particularly poor biologic understanding of chronic diseases such as diabetes, cardiovascular disease, chronic kidney disease, and hypertension, and this was closely related to the importance of disease expression through symptom complexes. For example, a 21 year old male stated, “*You know you have a disease because the body always has symptoms*.*”* As such, for chronic diseases, most of which have very few expressible symptoms, there were exaggerated expectations of cure, greater perceptions of treatment failure by biomedicines, and increased medical non-compliance which all led many participants to report transitioning to TMs even after being prescribed or given biomedicines. Results of the structured survey confirmed these findings. In the general population, chronic diseases were the second most common (15%) use for TMs exceeded only by daily symptomatic ailments (Fig 2a).

27yo M—*You know you are healed as you do not have to attend the hospital anymore because your symptoms have disappeared*.

53yo M—*Anything that stays in the human body for a long time without being cured*, *like amoebae and bilharzias [schistosomiasis] is a chronic disease*.

Medical Doctor—*For many patients with diabetes or hypertension*, *when their blood pressure or blood sugars are controlled*, *they believe they are cured*. *They come to follow-up in 2 to 3 months and see that their blood pressure and glucose are normal; therefore*, *they assume they are cured and stop their medications*. *The chronicity of these diseases they do not understand well*.

26yo F—*I attended the hospital and treatment was begun but with no success for a long time; instead other parts in my body began to swell*. *At that time I decided to discharge myself from the hospital*, *and my grandmother gave me local herbals which cured me*.

Medical Doctor—*Maybe it’s not treatment failure but the fact that some of the diseases are chronic*, *and patients keep on seeking a ‘cure’ for something that is a chronic disease*.

Herbal Vendor—*Traditional medicines work best for chronic disease*. *I can assure you if it is a chronic disease that within three months you can go for a test at the hospital and will find that you are healed*.

### Strong Cultural Identities

Ethnic identities are strong in Kilimanjaro even among urban communities and educated professionals. All of the MDs in the qualitative sessions reported frequent use of TMs, and urban participants with a professional occupation who completed the structured survey were more likely to report family tradition (RR = 1.80, p = 0.031) as an important reason for the use of TMs.

Elders (*wazee*) and family members were considered especially important sources of healthcare knowledge. When asked on the structured survey whom participants seek most commonly for healthcare advice, elders and family members was the second most common response (27%)(Fig 2b),and many participants explained during the qualitative sessions that they had adopted their practices from them. A 44 year old female stated, “*My family and I prefer not to go to hospitals*. *My grandparents taught us a lot (especially about plant roots) about healing and curing… my father still will not use any hospital medicines*,” and another female said, “*My daughter suffers from heart problems*. *I took her to an mzee* [elder] *in the rural Rombo district*. *Within two days my child was healed and remains cured to this day*.”

Distinct from their ethnic and family identities, participants also had strong national identities as Tanzanians. As such, many expressed a distinct ‘foreignness’ associated with biomedicines. The MDs agreed with this sentiment. One MD stated, “*The concept of taking pills on a daily basis is seen as a distinctly Western [American and European] thing*,” and a 53 year old man went as far as to say, “*Most of us believe that the Western people came here to undermine us and deter our local medicines*. *I think that they want to colonize us again*.” However, in most cases, the expression of this ‘foreignness’ was much more subtle and emerged as concerns over the side effects of biomedicines and their ingredients:

25yo M—*Our elders have taught us that hospital medicines have a lot of chemicals*, *and in my home area I know people who have used hospital medicines and had many side effects*.

23yo M—*I know someone who was using birth control pills*, *and she was infected with reproductive cancer as its side effects*.

65yo F—*People take traditional medicine to avoid hospital medicines which are full of chemicals that can affect their health*.

36yo F—*My friend will not use hospital medicines for anything because he does not know how those medicines are made*.

Herbal Vendor—*I remember that during our ancestor’s era there was no hospital*. *People were just using traditional medicines and women were having birth at home*, *but now you must go to hospital*. *Because of this*, *many diseases have erupted*.

### Health Status

An individual’s overall health status was a major factor in the decision to use TMs (Fig 1). Structured survey participants with a history of a chronic medical condition (diabetes, hypertension, heart disease, chronic swelling) were more likely to report TM use in the previous year (RR = 1.4, p = 0.043), and among those who were healthy (or perceived themselves to be healthy), the use of TMs was mostly limited to alleviating daily symptomatic ailments which in many instances were easily obtained from street-side herbal vendors (Fig 2a).

When sickness occurred, the chronicity, and the perceived severity of the disease influenced the decision to use TMs. In the qualitative sessions, a 36 year old female said,

*The frequency of traditional medicine use depends on the magnitude of the problem*. *For chronic problems people tend to use traditional medicines more frequently*. *This is opposed to “normal problems” for which people only take them when they feel sick*, *and they then stop afterwards when they no longer feel sick*.

Another female, a 44 year old, also said,

*The use of the traditional medicines depends on the extent of the problem*. *For example*, *when I have stomach problems I take traditional medicines*. *When I am healed I stop taking them immediately until I feel the abnormality again*.

In some instances, a dichotomy between ‘minor’ and ‘major’ health problems emerged. A 52 year old female said, “for smaller health problems people prefer traditional healers, and for the larger ones people prefer hospital services,” and a traditional healer said,

*I best treat diarrhea*, *foot swelling*, *waist tightening*, *poor masculine power*, *and cancer*. *Medical doctors*, *who are able to use various advanced testing kits*, *are able to treat more complicated problems than us who use only our naked eyes to diagnose patients*.

However, the distinction between minor and major health problems was not always clear. Some participants considered a health problem to be major when it produced severe, ongoing discomfort and incapacity while others did not consider a health problem to be major until experiencing a failure of TMs. In these instances, such as with chronic diseases and cancers that may be asymptomatic until advanced stages, the initial use of TMs was influenced by the other major constructs such as disease understanding and healthcare delivery; however, the ongoing use was strongly influenced by health status. This led them to attend biomedical clinics, but they frequently returned to TMs after experiencing a further decline in health status due to real or perceived treatment failure. This was also particularly prominent among participants with advanced or terminal diseases, and this emerged mostly in the form of seeking a miracle cure. A 58 year old male said, “*Stay with your God*, *but let us look for other alternative cures including traditional medicines or even witchcraft*. *This attitude is really prominent for people who have spent a lot of time in the hospital*.*”*

### Credibility of Traditional Practices

The credibility of traditional practices was closely related to cultural beliefs in TMs, yet it emerged as a separate major determinant (Fig 1). This credibility was influenced by factors distinct from cultural beliefs. Participants expressed concern over the lack of scientific validity, lack of appropriate dosing, lack of education, and lack of regulation among traditional healers and practices, but many of those same participants still held strong cultural beliefs in TMs and personally used them. A 53 year old female said, “*Herb Vendors or Traditional Healers may advise you to take one cup everyday but not specify the size of the cup in accordance with the age of the patient*,” and a 35 year old male stated, “*There is no scientific investigation that has been done to measure their efficacy*, *yet many people still find cures from them*.”

Traditional healers and herbal vendors themselves expressed the importance that credibility plays in their business. To increase the credibility of their medicines some vendors demonstrated their efficacy by mixing them with dyes to produce a colorful and visible reaction for customers, and other used these same reactions to demonstrate the potential harms of biomedicine. In the structured survey, when we asked participants why people most commonly use TMs, a perceived increased safety profile and effectiveness of TMs compared to biomedicine composed nearly half (45%) of the responses (Fig 2c).

All participants in the qualitative sessions discussed the numerous newspaper, radio, television, and billboard advertisements extolling the benefits of TMs. The healers and vendors additionally made extensive use of witness testimonials in the promotion of their services. A 27 year old male stated, “*It is a competitive market and the traditional healers advertise with a lot testimonials… we believe them because these testimonials are from patients who used hospital medicines and experienced side effects*.”

## Discussion

Reported use of TM is high in Northern Tanzania. It was used by people of all incomes in both urban and rural settings, and the most common reasons for use were daily symptomatic ailments and chronic diseases. More specifically, we identified five major determinants for its use: biomedical healthcare delivery, credibility of traditional practices, strong cultural identities, individual health status, and disease understanding.

Our work expands on previous studies across sub-Saharan Africa which have described these constructs to be important determinants of TM use among specific populations or conditions [7–14]. Biomedical healthcare delivery has been studied as a strong determinant of TM use especially in rural areas or among urban poor[10, 15, 16, 19, 22, 36]; however, we found that TM use extended to those with higher education levels, professional occupations, and across all ages in both urban and rural settings in Northern Tanzania. In other regions, disease understanding as a determinant of TM use has been studied primarily as a function of cultural belief models or disease characteristics such as an individual’s experience through the appearance and disappearance of symptoms [14, 15, 17]. In our study population, disease understanding was a complex composite interaction among healthcare delivery, strong cultural identities and beliefs, disease expression, and disease chronicity, and we found disease understanding to be an especially prominent determinant for TM use among participants with chronic diseases. This is consistent with explanatory models for chronic diseases in other countries across sub-Saharan African where chronic disease understanding was drawn on biomedical health knowledge, traditional cultural beliefs, and environmental factors as they relate to disease characteristics through symptom expression[16, 18, 20].

In our study, the credibility of traditional practices *vis-à-vis* the lack of monitored dosing, scientific evidence, and diagnostic testing was a strong determinant of TM use, and this is similar to other studies that have cited TMs as being viewed as “outdated” or “backwards” [14]. However, we found the perceived credibility of traditional practices was closely related to participants’ strong cultural identities, which in Tanzania included both ethnic and national identities. These dual identities were not only associated with a strong perception in the effectiveness of TM among many participants but also to biomedicine being viewed as distinctly foreign or different.

We also found that participants frequently transitioned between biomedicine and TM for numerous conditions. This traditional-biomedical doctor shopping occurred in both directions with people using both systems of care simultaneously, and it is consistent with other works in Tanzania and elsewhere that have observed concurrent biomedicine and TM use, frequent self-treatment with both TM and biomedicine, and high rates of doctor shopping even for conditions with strong links to TM like epilepsy [3, 8,12, 13, 37–39]. However, we found that traditional-biomedical doctor shopping also extended to chronic diseases especially as the individual’s health status changed or declined. As has been shown other sub-Saharan African countries with high rates of chronic diseases, this may be particularly important in Northern Tanzania where non-communicable diseases are a rapidly growing burden and TM use for chronic diseases is high [12,16,40–42].

Our study has many strengths. The application of insider-outsider coding to the inductive framework method ensured that all emerging themes were grounded in the data. The broader perspective provided by this technique allowed for finer coding of the data: nuances and cues that may have been overlooked by either coder alone were detected through a semi-dialectic process during review of the analytic memos and code index. Additionally, the mixed-methods design allowed for triangulation from multiple data sources and reproducible methods, and finally, the community-based sample allowed for results that are more generalizable across the regional population.

We also noted a few limitations. Our positionality as biomedical practitioners may have limited our ability to interpret results (researcher bias) about the underlying cultural framework leading to the use of TM, and as such, the qualitative and quantitative data collection were centered around TM use for disease categories that are derived from a biomedical perspective (e.g. NCDs and kidney disease can be considered biomedical constructs). Additionally, although we used insider-outsider coding, local non-medical surveyors and piloted questions to ensure content validity, reporting bias was still likely present. Participants may have been reluctant to answer truthfully regarding some topics, may have been more likely to report seemingly desirable information for others, or their statements may have been interpreted from the position of the researcher. As an example, we experienced divergence on the topic of disease-testing locations. In the qualitative sessions, participants, including the healers and vendors, strongly endorsed that most people attend hospital clinics for diagnostic testing only but prefer to seek treatment from traditional healers; however, on the structured survey nearly all participants responded that they do attend hospital clinics for diagnostic testing *and* treatment. The divergence seen on this topic of healthcare seeking behavior has been observed elsewhere [8], and in our case, it may have been due to a combination of reporting bias experienced on the structured survey and researcher bias due to our positionality as biomedical practitioners. However, it is consistent with prior work in Tanzania that showed traditional healers do often refer patients to biomedical facilities for diagnostic testing, and this suggests that the divergence we observed was more likely due to reporting bias on the structured survey alone [37].

In conclusion, we found that use of TM is high in Northern Tanzania and is especially frequent for symptomatic ailments and chronic diseases; further, people frequently transition between biomedical doctors and traditional healers, often obtaining treatment from both groups at the same time. We studied the users, frequencies, and reasons for TM use among a community-based sample in Northern Tanzania, and we described five major determinants of TM use which will be useful for formulating more effective local disease management programs that are more appropriately sensitive to TM practices.

## Supporting Information

S1 Appendix Moderator’s Guide for the Focus Group Discussions. (DOCX)

S2 Appendix Structured Survey Instrument for the use of Traditional Medicines (English and Swahili). (DOCX)

S3 Appendix Standard Operating Protocol (SOP) for Household Selection. (DOCX)
