# Supplementary material for: The Determinants of Traditional Medicine Use in Northern Tanzania: A Mixed-Methods Study
Source: PLoS One. 2015 Apr 7;10(4):e0122638. doi: 10.1371/journal.pone.0122638 (PMC4388565; doi:10.1371/journal.pone.0122638)
Supplement: S1 Appendix — (DOCX) [file pone.0122638.s001.docx]

**The Determinants of Traditional Medicine Use in Northern Tanzania: A Mixed Methods Study**

**Supplementary Appendix S1:**

**Focus Group Discussion (FGD) Moderator’s Guide**

John W Stanifer, MD, MSc^1,2^; Uptal D Patel, MD^1,3^; Francis Karia, MBA, MSc^4^; Nathan Thielman, MD, MPH^1,2^; Venance Maro, MD, MMed^4^; Dionis Shimbi, BS^4^; Humphrey Kilaweh, BS^4^; Matayo Lazaro, BS^4^; Oliver Matemu, RN^4^; Justin Omolo, PhD^5^; David Boyd, PhD ^2^

For the

Comprehensive Kidney Disease Assessment for

Risk factors, epIdemiology, Knowledge, and Attitudes (CKD AFRIKA) Study

1 Department of Medicine, Duke University; Durham, NC United States

2 Duke Global Health Institute, Duke University; Durham, NC United States

3 Duke Clinical Research Institute, Duke University; Durham, NC United States

4 Kilimanjaro Christian Medical College; Moshi, Tanzania

5 Tanga AIDS Working Group; Tanga, Tanzania

**Focus Group Discussion and Survey Pilot:**

Moderator’s Discussion Guide

GENERAL OUTLINE OF FOCUS GROUP

1. Seating/Refreshments
2. Introduction
3. Informed Consent
4. Ground Rules
5. Demographics Collection
6. Discussion
7. Survey Pilot
8. Conclusions
9. Compensation and Receipts
10. Team Debriefing

Groups: Females, ages 18 and older

Males, ages 18 and older

Facilitators: Moderator

Liver Translator/Transcriptionist

2 Research Assistants taking notes

Preparation: Ensure that all materials and supplies are available. Refreshments should be provided in the back of the room. Chairs should be arranged according to the seating chart in the Focus Group Standard Operating Procedures (SOP) Manual. Each chair should be clearly numbered.

Questions: Short questions, no long pre-ambles, clear instructions

It is important to build rapport with the participants but to also present yourself as a researcher rather than a friend.

Clarify: lack of knowledge/experience is not a problem

We do not care what should be, but what people really feel and do

Moderator to initially ask question verbatim

- Break into influence (What prompted you?) and attributes (What features?)
- Use follow-up questions and probe questions to ‘dig deeper’
- Avoid leading or judgmental statements
- General before specific
- Positive before negative

1. **Seating and Refreshments**
   - - Participants will receive a Numbered Tag to display on their shirts when they enter the room. *Please ensure that the Tag is clearly displayed on each participant’s shirt.*
     - The Numbered Tag that each participant receives will serve as their study ID number as well as indicate which chair he/she is to sit in. *It is the responsibility of the research team to make sure that this number is recorded in the enrollment logbook and demographics form.*
     - Welcome each participant to the refreshments.
     - The transcriptionist should use this time to fill out the enrollment logbook. Approach each participant individually to fill out the logbook. *Ensure that the Tag Number is recorded accurately in the logbook.*
     - Ask each participant to turn off his/her cell phone.
     - When everyone has received a Numbered Shirt Tag, been enrolled into the Enrollment Logbook, and been seated then move on to the Introduction.
2. **Introduction**

**Introduce staff attending the focus group**

- Moderator
- Transcriptionist
- Research Assistants taking notes

**Introduce Focus Group Purposes and Objectives** (the following paragraph should be read aloud verbatim)

Karibuni katika mjadala wa kikundi kwenye utafiti wetu. Mjadala huu ni sehemu ya utafiti mkubwa unaohusiana na kutafuta kufahamu utaratibu unaohusiana na matumizi ya asili ya mitishamba, na madawa ya asili kwa badhi ya watu wa Kilimanjaro Pia tunataka kufahamu baadhi ya mambo yanayohusiana na uangalizi wa magonjwa sugu kama magonjwa ya figo kama yanavyofahamika kwenye hii jamii. Zaidi ya hapo, tunapenda kuhakikisha kuwa savey yetu inaeleweka na wakazi wa hapa kabla hatujaanza kuitumia. Utafiti huu unafadhiliwa na Chuo Kikuu cha Duke na chuo cha Tumaini hapa KCMC.

Nyie wote mpo hapa kwasababu mmejitolea kushiriki katika mjada wa ukweli na wazi kuhusu hizi mada. Fidia ya fedha itatolewa kwenu baada ya mjadala huu. Katika majadiliano ya leo, tutawauliza maswali kadhaa tofauti kuhusu mnavyofikiri kuhusu matumizi ya dawa za asili, mambo ya maadili, na savei yetu. Tafadhali kumbuka kuwa hakuna majibu yasiyo sahihi au yaliyo sahihi. Tunapendelea zaidi kujifunza namna mnavyofikiri.

Tutakuwa tunarekodi (chukua sauti) mjadala wa leo kwasababu tunapenda tusipoteze mawazo yenu. Hakuna majina yatakayohusishwa katika taarifa yoyote, na michango yenu itakuwa siri. Kabda ya kuanza sehemu ya mjadala wa leo ni lazima kila mmoja aweke sahihi katika fomu ya idhini ya ridhaa ambayo inaelezea haki zenu kama washiriki wa utafiti. Haki zenu kama washiriki wa utafiti huu ni muhimu. Tunapenda kupitia fomu ya idhini ya ridhaa pamoja na kuwaomba kuweka sahihi. Kuna nakala mbili, moja ambayo tutaihifadhi na nyingine ambayo itakuwa yakwenu.

(English translation) – for moderator’s use only (not to be read aloud)

*Welcome to the live group discussion of our research study. This discussion is part of a larger study related to exploring and understanding practices related to the use of natural, herbal, and traditional medications among the general population of Kilimanjaro. We also want to explore some issues related to the management of chronic disease such as kidney disease as understood by the local population. Additionally, we want to ensure that our surveys are easily understood by the local population before we employ them. This study is sponsored by Duke University and Tumaini College at KCMC.*

*All of you are here because you volunteered to participate in an open and honest discussion about these topics. Monetary reimbursement will be provided to you at the end of the session. During today’s discussion, we will ask you a number of different questions about your thoughts on the use of herbal medications, ethical issues, and our surveys. Please keep in mind that there are no right or wrong answers. We are only interested in what you think.*

*We will be recording today’s session because we do not want to miss any of your comments. No names will be included in any reports, and your comments are confidential. Before we begin today’s session, you must sign an informed consent form which describes your rights as research participants. Your rights as a participant in this research study are important. We would like to review the Informed Consent Form and ask you to sign it. There are two copies, one which we will keep and the other is yours.*

1. **Informed Consent**

- Distribute and review the informed consent form

- Read the informed consent form verbatim

- Before each new heading ask if there are any questions.

- Research Assistants ask each participant individually whether he/she is ready to sign the form.

- Update the Enrollment Logbook and ensure that the Tag Number is recorded on the Informed Consent Forms.

- The participant is offered a copy of the consent form.

- Collect signed informed consent from all participants before continuing.

**D. Ground Rules** (read the following paragraph aloud)

Tunategemea kuwa mjadala utadumu kwa kama masaa 2. Tutakuwa na mapumziko baada ya kama saa 1 na unakaribishwa viburudisho wakati wowote wa mjadala.

Usiri – Kila mmoja anayeshiriki katika mjadala huu wa kikundi atakuwa anashiriki kutoa maoni na mtazamo mbele ya wengine katika kikundi hiki. Kama watafiti, tunawahakikishia kuwa hatutajadili maoni yako binafsi na mwingine yeyote asiyehusika na utafiti huu.Tunawaomba nyie wote kufuata utaratibu. Tafadhali tuheshimu kuwa wote tunazungumza ukweli na tafadhali usiyaseme yaliyosemwa na wengine. Kwa maneno mengine, yanayosemwa hapa, yabakie hapa.

Hakuna majibu yaliyo au yasiyo sahihi. Tunapenda kila mmoja kujisikia vizuri kushiriki taarifa nasi leo hii. Maoni ya kila mmoja yanathaminiwa. Hakuna jibu sahihi au lisilo sahihi, ni utofauti wa mitazamo kwa vipengele tofauti. Maoni yako wakati wa mjadala hayataathiri uhalali wako kupokea fidia mwisho wa mjadala; uhitaji pekee wa kupata fidia ni kuwa utahudhuria muda wote wa mjadala na kushiriki kikamilifu.

Tafadhali sikiliza kwa umakini na uheshimu wengine wakiongea. Usimwingilie mwongozaji akiongea na tafadhali epuka kuzungumza chinichini. Iwapo unataka kufuatilia chochote ambacho mwingine amesema kwa kukubali, au kutoa mfano hapo tafadhali jisikie huru kufanya hivyo. Jisikie huru kujadiliana na wengine kuhusu maswali haya.

Tunapenda kuhakikisha kuwa tunasikiliza kutoka kwenu wote leo. Iwapo itawezekana, wakati ukianza kuongea, tafadhali sema namba yako kwani hii inasaidi kwenye unasaji wetu wa sauti.

Wajibu wetu kama wawezeshaji ni kuongoza mjadala na kusaidia kuendeleza majadiliano. Kazi yetu pia ni kusikiliza chochote kutoka kwenu wote, ili tuweze kuwauliza maswali yaliyoandaliwa kufanya hivyo. Tunaweza kuita kila mmoja wenu kwa namba zenu iwapo tutahitaji.

Tutaanza kwa kuwasaidia kujaza fomu za taarifa binafsi. Baada ya kukamilisha fomu hizi tutanza kunasa sauti (kurekodi).

Je, kuna yeyote mwenye swali?

(English translation) – for moderator’s use only (not to be read aloud)

*We anticipate that the session will last a few hours. We will take periodic breaks and you are welcome to refreshment at any time during the session.*

*Confidentiality – Everyone participating in this group will be sharing comments and views in the presence of other members of this group. As researchers, we assure you that we will not discuss your personal comments with anyone not involved with this research. We ask that you all please follow the same rules. Please respect that we are all speaking honestly and please do not share what is said with others – in other words, what is said here, should stay here.*

*There are no right or wrong answers. We want everyone to feel comfortable sharing information with us today. Everyone’s opinion is valued. There are no right or wrong answers, only differing points of view. Your comments during the discussion will not affect your eligibility to receive that compensation at the end; the only requirement for the compensation is that you are present for the entire discussion and actively participate.*

*Please listen actively and respect others when they are talking. Do not interrupt the speaker and please avoid side conversations. If you want to follow up on something that someone has said by agreeing, disagreeing, of giving an example then please feel free to do that. Feel free to have a conversation with one another about these questions.*

*We want to make sure that we hear from all of you today. Please speak up but only one person should talk at a time. If possible, when you start speaking, please state your number as this helps with our audio recordings.*

*Our role as moderators is to guide the discussion and help move the conversation along. Our job is also to hear something from everyone, so we may ask you questions designed to do that. We may call on each of you by your number if we need to.*

*We will first start by helping you to fill out a basic demographics form. After you have completed these forms we will begin recording.*

*Does anyone have any questions?*

1. **Demographics Form**

- Hand out the demographic form to each participant
- Read aloud each part of the demographics form and give each participant enough time to fill out each question before moving on to the next.
- The research assistants should walk around the room to ensure that each participant understands and is able to fill out the form entirely. *Please make note that some participants may not be able to read and write. These participants will require extra attention and may require that the research assistant fills out the form for them.*
- Collect the forms after each participant has filled them out. Make note that the Tag Number is marked on the form and matches that on the participant’s shirt and in the enrollment logbook.
- Briefly review the forms to ensure that they have all been fully completed.

1. **Discussion** *(Turn on recording at this time)*

***Note to Moderator:*** *This guide should help inform the discussion and includes key topics and questions; the flow of the group does not have to follow this guide exactly*

| **Time** | **Question** |  | **Notes** |
| --- | --- | --- | --- |
| 35 min | Welcome  Consent  Ground Rules |  | Scripted as noted above |
| 10 min | Demographics |  | Scripted as noted above |
|  | ***Turn on Audio Recorded*** |  |  |
| 5-8 min | - - - 1. Baada ya kufikiwa mara ya kwanza na timu yetu ya utafiti, nini kilikuja kichwani kwako.   *When you were first approached by our research team what came to your mind?* |  | Ice breaker. Get everyone to talk early. Not critical to analysis. Avoid power/status-relevant questions.  KUMBUKA: “Hakuna jibu sahihi au lisilo sahihi”  *REMINDER: “There are no right or wrong answers.”* |
| 20 min | - - - 1. Kama tulivyokwambia awali, mjadala huu wa kikundi ni kuhusu asilia na madawa ya asili.   Ninapenda kusikia kutoka kwa kila mmoja wenu kuhusu uzoefu wenu na madawa ya asili?   - - - - 1. Unafahamu watu wanaoitumia?         2. Miti shamba/Dawa gani za asili umesikia habari zake?         3. Kuna faida kwa kuzitumia?         4. Je zinatumika sana?   *As we told you earlier, this focus group is about natural and traditional medicines.*  *I would like to hear from each of you about your experiences with herbal medications?*  *Do you know people who take them?*  *What herbals have you heard about?*  *Are there benefits of taking them?*  *Are they common?* |  | Encourage a response from each person. Use probing.  KUMBUKA: “Dawa za asili/miti shamba zinaweza kuhusisha majani, vidonge, vitamin, dawa za kufukiza, chai na supu ambazo zinatumika kutibu afya na matatizo ya kujiweza.  *REMINDER: “Natural or traditional medicines may include herbs, drugs, vitamins, inhalations, teas, foods, creams, lotions, potions, and soups that are used to treat health and wellness problems.”*  Try to get specifics on how often people take them, e.g. daily, weekly, monthly, etc. |
| 20 min | - - - 1. Ni kwasababu zipi unafikiri watu wanatumia madawa ya asili.          1. Je, watu wanatumia kupata bahati?          2. Je gharama au upatikanaji ni sababu ya watu kutumia   *For what reasons do you think people take natural or herbal medications?*  *Do people take them to improve luck? Or ward off curses?*  *Is cost or access a reason that people take them?* |  | Start by speaking in 3^rd^ person before asking in 2^nd^ person (“do you think people take them?” rather than “do you take them?”)  Unaweza kudodosa kwa kusema: tumesikia kuwa watu kwakawaida wanatumia kwa ugumba, homa, macho “mabaya”, na kuumwa kichwa.  *May probe by saying: we have heard that people commonly take them for infertility, fever, “evil” eyes, and headache.*  MDs and Hospital may be too far away or may be too expensive. |
| 10 min | - - - 1. Je, kuna matatizo ya afya ambayo yanatibika vizuri zaidi kwa mganga wa kienyeji/asili na mengine kwa daktari wa hospitali?          1. Matatizo gani yanatibiwa vizuri na mganga wa asili?          2. Yapi yanatibiwa vizuri zaidi kwa daktari wa hospitali?   *Are there health problems that are treated best by traditional healers and some that are best treated by Medical Doctors (MD)?*  *What problems are best treated by traditional healers?*  *Which ones are best treated by MDs?* |  | Kutokuwa na uwiana kwa watu ‘kujiweza’ kunaweza tu kusitibiwe na vidonge-kunaweza kuhitaji utaalamu wa mganga wa asili.  *An imbalance of personal ‘well being’ cannot just be treated by a pill – may require the expertise of a traditional healer?* |
| 10 min | - - - 1. Je, unafikiri kuwa watu wanatumia madawa asili kukwepa kudhaniwa kuwa wagonjwa?          1. Je, watu ni wawazi kuhusu utumiaji wao wa dawa za asili?          2. Unadhani watu watajibu maswali ya savei kwa ukweli kuhusu madawa asili?          3. Nini kinaweza kusababisha mtu kushutumiwa kuwa mchawi?   *Do you think that people take traditional medications to avoid being perceived as sick?*  *Are people open about their usage of traditional medicines?*  *Do you think people will answer a survey about traditional medicines honestly?*  *What would cause someone to be accused of withcraft?* |  | Stigma associated with going to an MD such as perceived as having HIV or being sick.  Try to understand the distinction between traditional/herbal medications, rituals/divinations, and witchcraft.  Jaribu kuelewa tofauti kati ya dawa asili, uchawi, mitambiko na uaguzi  Je watu huwa wanatumia mitishamba kudhuru watu mfano: kama sumu  *Do people ever use herbals to inflict harm, i.e. as a poison?* |
|  | **Sasa tunabadili mada na kulenga kwenye magonjwa sugu ya figo.**  ***Now we are going to shift focus to Chronic Diseases and Kidney Disease*** |  |  |
| 25 min | - - - 1. Umewahi kudhani kuwa unaweza kupata ugonjwa wa figo?          1. Nini kilikufanya au kinaweza kukufanya ufukiri kuwa una matatizo ya figo?   *Have you ever thought that you might have kidney disease?*  *What prompted or would prompt you to think that you have kidney problems?*   - - - 1. Unaweza kuwa na wasiwasi kuhusu hadhi yako katika jamii iwapo utakutwa una ugonjwa wa figo?   *Would you be worried about your reputation in the community if you found out you have kidney disease?*   - - - 1. Je, dawa za asilia zinafanyakazi kama tiba kwa ugonjwa wa figo?   *Would traditional medicines work as a form of treatment for kidney disease?* |  | Watu wanaelewa nini kuhusu ugonjwa wa figo? Je wanadhani kuwa ugonjwa wa figo ni sawa na ugonjwa wa njia ya mkojo (UTI)?  *What is people’s understanding of kidney disease? Do they think that kidney problems are the same thing as a urinary tract infection?*  Very important question. We are trying to gauge if people commonly take herbals when they find out they have kidney disease. |
|  | ***Hand out Traditional Medicine Surveys*** | | |

1. **Traditional Medicine Survey Pilot**

- Distribute the Traditional Medicine Survey. Ensure that each participant has a pen as well.

Madhumuni ya sehemu hii ni kujaribu au kupima madodoso yetu kabla ya kuyatumia kwa kiwango kikubwa. Ni muhimu kwetu kuwa unaonyesha ugumu wowote, kutojichanganya, au maneno yasiyo na maana

Tumia kalamu uliyopewa kuweka alama kwenye maswali au maneno ambayo huyaelewi. Iwapo huelewi swali, tafadhali weka alama au zungushia swali na tutayapitia yote mwishoni. Tafadhali usijibu maswali ambayo huyaelewi. Iwapo unaelewa lakini ukweli hujui jibu, jisikie huru kuweka alama kwenye kisanduku cha “sifahamu” au “sina hakika”

Tutasimama baada ya kila maswali machache kujadili tuliyokwisha kukamilisha na kupata maoni yenu. Kumbuka, hakuna majibu yalio au yasio sahihi kwa maswali haya. Tunahitaji ukweli wenu na mrejesho nyuma.

(English translation) – for moderator’s use only (not to be read aloud)

*The purpose of this session is to pilot or test-out our questionnaires before we use them on a larger scale. It is important to us that you express any difficulties, confusion, or words that do not make sense.*

*Use the pen given to you to please mark questions or words that you do not understand. If you do not understand a question then please mark or circle that question and we will review them all at the end. Please do not answer questions that you do not understand. If you understand but genuinely do not know the answer then feel free to then mark the ‘don’t know’ or ‘unsure’ box.*

*We will stop after every few questions to discuss what we have covered and get your thoughts. Remember, there are no right or wrong answers to these questions. We are only seeking your honest opinion and feedback.*

Traditional Medicine SURVEY

- Read the instructions of the Survey as if you were administering it.
- Read each question aloud. Ensure that each person has adequate time to answer the question.
- Each question should be read smoothly and be easily understood. If the participants appear confused or hesitant to answer then find out why.
- As you read the questions, each team member should be observing the reactions and attributes of the participants.
- All members of the team should watch the participants complete each question. Watch for hesitation, erasures, or skipped questions. Please seek verbal feedback on an individual level and please take note of what you observe.
- It is okay to clarify a question but do not try to point or lead the participants toward one particular answer.
- Try to elicit a response from each individual. This may require calling on specific persons.
- After each question ask the following to the group:
  - - 1. Kuna yeyote amepata shida na swali?
      2. Je, kuna maneno yoyote ambayo ni magumu kuelewa au hayaleti maana au kuonekana mageni?
      3. Je, kuna dhana zozote au mawazo ambayo unafikiri kuwa hayana maana au mageni?
      4. Muombe mshiriki “kurudia kutamka” swali
      5. Kuna chochote ambacho kinaweza kuwa kigumu au cha binafsi sana kwa yeyote kuweza kujibu mbele ya mdahili au wanakaya wengine?
      6. Una mapendekezo yoyote ambayo yanaweza kusaidia kufanya swali hili kuwa boza zaidi? (Mpangilio, matamshi, zuri/baya, mambi binafsi, kutumika)
      7. (For questions 2, 4, 5, 6 only) Kuna uchaguzi wowote ambao ulitaka kujibu lakini hukuhusishwa? Kuna yoyote ambayo tumeyahusisha lakini unadhani yasingehusishwa?

1. *Did anyone have difficulty with the question?*
2. *Are there any words that are hard to understand or do not make sense or seems strange?*
3. *Are there any concepts or ideas that you think do not make sense or seem strange?*
4. *Ask a participant to “parrot back” the question.*
5. *Is there anything that might be difficult or sensitive for someone to answer in front of an interviewer or other family member?*
6. *Do you have any suggestions that could make this question better? (Order, wording, good/bad questions, sensitive matters, applicability)*
7. *(For questions 2, 4, 5, 6 only) Are there any choices that you wanted to answer but were not included? Are there any that we did include but you felt should not be?*

**H. Conclusion/Wrap-Up**

- Turn off the tape recorder at this time.

Nawashukuru sana kwa ushiriki wenu katika mjadala wa kikundi wa leo. Mawazo yenu na maoni yamekuwa ya maana sana kwetu. Kabla ya kumaliza sehemu ya leo, kuna yeyote mwenye swali au manung’uniko kuhusu tuliyoyajadili hapa? Pia, tafadhali jisikie huru kufurahia viburudisho zaidi kabla ya kuondoka.

Iwapo una maoni yoyote au manung’uniko kuhusu haki zako kama mshiriki wa utafiti tafadhali rejea kwenye taarifa za mawasiliano katika muongozo wa Majadiliano. Sasa tutatoa fidia kwa muda wenu.

(English translation) – for moderator’s use only (not to be read aloud)

*Thank you for your participation in today’s focus group discussion. Your thoughts and opinions have been very valuable to us. Before we end today’s session, does anyone have any questions or concerns about what we discussed here today? Also, please feel free to enjoy some more refreshments before you leave.*

*If you have any comments or concerns about your rights as research participants then please refer to the contact information in your Discussion Guide. We will now hand out the compensation for your time.*

**I. Compensation**

- Envelopes containing the cash reimbursement should be handed out individually.
- To receive the cash reimbursement each participant must sign or thumbprint the Receipt form. Please ensure that the Tag Number is recorded at the top of the compensation form.
- Two copies of the receipt should be obtained: One for our records and one for the participant to keep.
- After the participant signs the receipt, please record that he/she received the compensation in the Enrollment Logbook.
- After the Enrollment logbook has been updated, all information has been verified to be correct, and all forms have been collected the participant can be dismissed.

**J. Debriefing**

- After everyone has left, gather the entire research team for a debriefing session.

- This is the time for reviewing the recordings, looking over the notes, and discussing how the session went.

- The moderator or study coordinator will lead the session and fill out the Debriefing Discussion Form.
